# 

**Published:** 1908-12

**Authors:** 


					CONTENTS.
Page
Recent Work on Leukaemia and some Allied Affections.
By J. Michell Clarke, M.A., M.D.Cantab., F.R C.P.
289
The Causation of certain Mid-diastolic Murmurs.
By Carey Coombs, M.D. Lond., M.R.C.P.
312
Notes on Cases.
By T. Carwardxne, M.S. Lond., F.R.C.S.
3i7
Dilatation of Posterior Cerebral Vesse's with Basal Hemorrhage
in a Girl aged sixteen.
By T. M. Carter, M.D.
322
A Medical Naval Volunteer on Duty at Quebec, July, 1908.
By W. Kenneth Wills, M.B., B.C.Cantab.
327
Progress of the Medical Sciences :
Medicine.
J. M. Fortescue-Brickdale, M.A., M D., B.Ch. Oxon.
335
Surgery.
James Swain, M.D., M.S. Lond.
339
Reviews of Books
343
Editorial Notes
357
Notes on Preparations for the Sick
364
The Library of the Bristol Medico-Chirurgical Society ... ... 366
Meetings of Societies  ^ OF COAy>
3^9
Obituary Notices
Local Medical Notes
S OftO'1 A
r

				

